# Brown Algae Polysaccharides Alleviate Diquat-Induced Oxidative Stress in Piglets and IPEC-J2 Cells via Nrf2/ARE Signaling Pathway

**DOI:** 10.3390/ani15040559

**Published:** 2025-02-14

**Authors:** Chunjie Hou, Zirou Yu, Chenyu Shi, Ya Huang, Hu Liu

**Affiliations:** College of Animal Science & Technology, China Agricultural University, No. 2 Yuanmingyuan West Road, Beijing 100193, China; houchunjie@cau.edu.cn (C.H.); yuzirou0828@cau.edu.cn (Z.Y.); scyshichenyu@163.com (C.S.); huangya@cau.edu.cn (Y.H.)

**Keywords:** brown algae polysaccharides, piglets, IPEC-J2 cells, antioxidant capacity, Nrf2/ARE signaling pathway

## Abstract

This study investigated the effect of Brown algae polysaccharides (BAPs) on diquat-induced oxidative stress in piglets and IPEC-J2 cells through Nrf2/ARE signaling pathway. In the in vivo model, 24 male piglets of the Duroc × Landrace × Large White breed were selected and divided into 4 groups (n = 6), including the CON group (basal diet), DIQ group (10 mg/kg Diquat), BAP group (1000 mg/kg BAP), and BAP+DIQ group (1000 mg/kg BAP + 10 mg/kg Diquat). Compared with the DIQ group, BAP improved growth performance and the BAP+DIQ group reduced the levels of IL-1β, IL-6, TNF-α, and DAO in plasma, increased VH and VCR, improved jejunal tissue morphology, decreased MDA levels, and increased T-AOC (*p* < 0.05). Additionally, the BAP+DIQ group elevated mRNA levels of *ZO-1*, and enhanced the protein levels of Occludin, Claudin1, CAT, SOD1, and HO-1 (*p* < 0.05). In the in vitro model, the BAP+DIQ group decreased MDA levels, increased T-AOC, elevated mRNA levels of *ZO-1*, *CAT* and *SOD2*, as well as protein levels of Claudin1, SOD1, HO-1, and total Nrf2 compared with the DIQ group (*p* < 0.05). Furthermore, BAP increased nuclear Nrf2 protein levels, and promoted the translocation of Nrf2 from the cytoplasm to the nucleus compared with the DIQ group (*p* < 0.05). In conclusion, BAPs are crucial for enhancing piglets’ antioxidant capacity via Nrf2 pathway activation. These findings highlight BAP’s potential as a natural feed additive to mitigate oxidative stress and improve overall health in piglets. Further research is warranted to explore BAPs as a dietary supplement to support gut health and reduce oxidative stress.

## Simple Summary

Oxidative stress caused by weaning is widespread in pig breeding and poses a serious threat to the health of piglets. As a feed additive, Brown algae polysaccharides (BAPs) have a good application prospect and have antibacterial, anti-inflammatory, antioxidant, and antiviral activities. The present study was conducted to investigate the effects of BAP on oxidative stress in piglets. The results showed that BAPs alleviated the oxidative stress damage caused by diquat and improved the antioxidant capacity and intestinal barrier capacity of weaned piglets and IPEC-J2 cells. It is worth noting that BAPs also promoted the translocation of Nrf2 from the cytoplasm to the nucleus. These results provide a new way to illustrate the mechanism of BAPs alleviating oxidative stress in piglets.

## 1. Introduction

Weaning is one of the critical stages in the growth and development of piglets. Currently, most large-scale pig farms in China wean pigs at 19 to 25 days of age to improve sow reproductive efficiency and annual productivity [1]. During the weaning process, changes in factors such as mother–piglet separation, transportation, environment, and diet can induce oxidative stress syndrome in weaned pigs [2]. This severely impacts the immune function and growth performance of weaned pigs [3], which significantly reduces production efficiency and increases economic losses for the livestock industry [4,5].

Oxidative stress leads to a significant increase in free radicals and decreases the organism’s ability to eliminate them, which disrupts the dynamic balance between oxidation and antioxidant reactions [6]. Increasing evidence suggests that oxidative stress induces inflammatory responses in the intestines of weaned pigs [7] that damage intestinal morphology and functions, such as barrier integrity, nutrient absorption, and immune responses [8]. Farmers often use antibiotics to alleviate diarrhea and promote growth. However, issues such as antibiotic resistance and drug residues pose significant threats to animal and public health [9,10]. Consequently, China completely banned the use of antibiotics in weaned pig diets since 2020. Oxidative damage caused by early weaning stress in weaned pigs requires urgent mitigation. Therefore, finding novel antibiotic alternatives has become imperative.

Natural products derived from the ocean have excellent bioactivity. These attributes make ocean-derived products a valuable reservoir of new bioactive compounds, offering opportunities to discover drugs with unparalleled chemical novelty [11]. In recent years, Brown algae polysaccharides (BAPs) have garnered attention for their potential to protect the gut health of humans and animals. Brown algae polysaccharides are collectively referred to as polysaccharides isolated and extracted from brown algae, which mainly include alginate, fucoidan, and laminaran [12]. Brown algae polysaccharides contain numerous bioactive secondary metabolites and exhibit a variety of biological activities, such as anti-cancer, antibacterial, anti-inflammatory, antioxidant, and antiviral properties [13,14]. However, there is limited information on specific pathways through which BAPs improve gut health, particularly regarding their protective effects on intestinal barriers and enhancement of antioxidant capacity in piglets.

The present research aimed to utilize diquat-induced oxidative stress in piglets and IPEC-J2 cells to investigate the possible protective effect of BAPs on intestinal barrier function and reactive oxygen species levels.

## 2. Materials and Methods

### 2.1. Piglet Experiment

#### 2.1.1. Design of Experiment

In this 28-day trial, 24 male piglets of the Duroc × Landrace × Large White breed were selected, with initial body weights ranging from 8.30 kg ± 0.40 kg. The selected piglets were randomly divided into four groups (n = 6), including the CON group (basal diet), DIQ group (basal diet + 10 mg/kg Diquat), BAP group (1000 mg/kg BAP diet), and BAP+DIQ group (1000 mg/kg BAP diet + 10 mg/kg Diquat). The content of the diquat was 10 mg/kg according to previous studies (intraperitoneal injection, diquat dibromide monohydrate, 6385-62-2, Aladdin Co., Shanghai, China) [15], and the concentration of BAPs (purity ≥ 98%, Xi’an Deshengyuan Biotechnology Co., Ltd., Xi’an, China) used in this experiment was 0.1% according to previous studies [16]. The basal diet formulation was designed in accordance with the NRC (2012) nutritional requirements for piglets [17]. The composition and nutrient levels in the diets are shown in Table 1. The 24 piglets were individually housed in metabolic cages measuring 1.5 × 0.7 × 1.2 m, with unrestricted access to water and feed throughout the 28-day experiment. The environmental conditions were set to maintain a room temperature of 22–24 °C, relative humidity between 57 and 67%, and a 12 h light/dark cycle.

On the 21st day of the trial, piglets from the CON group and BAP group were intraperitoneally injected with sterile saline. Other groups were challenged with diquat. Throughout the experiment, daily feed consumption for each piglet was meticulously documented, and their weights were measured at 8 am on the 21st and 28th days of the study.

#### 2.1.2. Sample Collection

Upon conclusion of the trial, blood samples were collected via blind puncture of the jugular vein after a 12 h fasting using EDTA anticoagulation tubes. Following collection, the blood samples were subjected to centrifugation at 3000× *g* for a duration of 10 min at a temperature of 4 °C to separate the plasma. The plasma was then kept at −20 °C for further investigation. Subsequently, the piglets were electrically stunned and slaughtered, and the abdomen was opened immediately to collect segments of the jejunum and colon. About 2 cm segments of the middle of the jejunum and colon were isolated, gently flushed with ice-cold sterile saline, and then fixed in a 4% paraformaldehyde solution (40% formaldehyde solution dissolved in phosphate-buffered saline (PBS)) and stored at 4 °C for histological analyses. Jejunal tissue from the remaining intestinal segments were collected in freezing tubes, frozen rapidly in liquid nitrogen, and stored at −80 °C for further analysis.

#### 2.1.3. Morphological Examination of Intestinal Tissue

After fixation with a 4% paraformaldehyde solution, intestinal morphology was estimated according to a previous method [18]. Briefly, segments of the jejunum were dehydrated through a series of graded ethanol solutions, embedded in paraffin, sectioned into 5-micrometer-thick cross-sections using a microtome, and subsequently stained with hematoxylin and eosin. Sections on the slides were then enclosed with cover slips and cedar oil. Images were captured using a microscope (Olympus, BX-51, Tokyo, Japan) and analyzed for villus height (VH) and crypt depth (CD) using Image-Pro Plus software (Media Cybernetics, Rockville, MD, USA, Version 7.0). The villus-to-crypt ratio (VCR) was calculated as the villus height divided by the crypt depth.

#### 2.1.4. Measurement of Inflammatory Mediators and Gut Permeability Markers

Concentrations of plasma D-lactic acid (D-LA), diamine oxidase (DAO), interleukin-1β (IL-1β), interleukin-6 (IL-6), tumor necrosis factor-α (TNF-α), and interleukin-10 (IL-10) were quantified using enzyme-linked immunosorbent assay (ELISA) kits (Nanjing Jiancheng Bioengineering Institute, Nanjing, China). All protocols were conducted strictly following instructions from the manufacturer.

#### 2.1.5. Measurements of Antioxidant Capacity

Frozen jejunal samples (0.1 g) were weighed and rinsed in ice-cold saline before being placed into a 2 mL homogenizing tube. Nine times the volume of normal saline was added to a homogenate tube at a ratio of weight (g) to volume (mL) = 1:9. Samples were homogenized 3 to 4 times (60 Hz, 60 s each) using a tissue homogenizer. The lysate was centrifuged at 12,000× *g* for 10 min at 4 °C to obtain supernatant. The levels of total antioxidant capacity (T-AOC) and malondialdehyde (MDA) in jejunal tissue were assessed in accordance with the manufacturer’s instructions (Nanjing Jiancheng Bioengineering Institute, Nanjing, China). The absorbance of the reaction mixture at 405 nm and 532 nm was finally determined using a UV-Vis spectrophotometer. Measurements were performed in triplicate to ensure accuracy and reproducibility.

#### 2.1.6. RNA Extraction and Quantitative Real-Time PCR (qRT-PCR)

Total RNA was extracted from the jejunum using a TRIzol Reagent (TaKaRa, Beijing, China). RNA concentration and purity were measured by Nanodrop spectrophotometer (Beckman Coulter, DU800, Brea, CA, USA). Reverse transcription and amplification were performed with a PrimeScript RT Kit with gDNA Eraser (TaKaRa, Beijing, China). TB Green Premix Ex Taq (TaKaRa, Beijing, China) was used for qRT-PCR on a real-time fluorescence quantitative system (Thermo Fisher Scientific, Waltham, MA, USA), with primers listed in Table 2. Glyceraldehyde-3-phosphate dehydrogenase (GAPDH) was selected as the housekeeping gene, and the target gene expression was analyzed using the comparative Ct (2^−ΔΔCt^) method [19].

#### 2.1.7. Protein Extraction and Western Blot

Protein was extracted from the jejunum samples using RIPA buffer containing PMSF protease inhibitor (Beyotime Biotechnology, Shanghai, China) and quantified using a BCA protein assay kit (Beyotime Biotechnology, Beijing, China). Sodium dodecyl sulfate-polyacrylamide gel electrophoresis (SDS-PAGE) separated denatured proteins, which were transferred to polyvinylidene fluoride (PVDF) membranes. After blocking with 5% milk, the membranes were probed with primary (1:1000, Proteintech, Rosemont, IL, USA) and secondary (1:10,000, Cell Signaling Technology, Beverly, MA, USA) antibodies. Proteins were detected with an Odyssey Clx fluorescence imaging system (LI-COR, Lincoln, NE, USA), and bands were quantified using ImageJ (Version 1.54m). For original images and antibody information of the experiment, see Supplementary Materials.

### 2.2. Cell Experiments

#### 2.2.1. IPEC-J2 Cell Cultures

Cells were cultured in a DMEM/F-12 medium supplemented with 10% fetal bovine serum and 1% antibiotics (Gibco Laboratories, Life Technologies Inc., New York, NY, USA) at 37 °C in a 5% CO_2_ atmosphere. The cells were seeded at a density of 1 × 10^5^ cells/mL in 6-well plates (Corning, Corning, NY, USA) and incubated overnight. Subsequently, the cells were treated with BAP and DIQ either individually or in combination for 8 h. After treatment, the cells were collected for further detection and analysis.

#### 2.2.2. Treatments for Oxidative Stress and BAPs in IPEC-J2 Cells

IPEC-J2 cells were plated in a 96-well plate and reached 70–80% confluency after approximately 24 h. After replacing the media, diquat solutions (0, 100, 200, 300, and 400 mg/L) were added to the wells (six replicates per treatment). Two hours later, the media was replaced again, and cell viability was assessed using a Cell Counting Kit-8 (CCK-8; Solarbio Life Sciences, Beijing, China) according to the manufacturer’s instructions.

In an independent trial, IPEC-J2 cells were seeded in a 96-well plate and reached 50–60% confluency after approximately 12 h. After replacing the media, BAP solutions (0, 500, 1000, 1500, and 2000 mg/L) were added to the wells (six replicates per treatment). Six hours later, the media was replaced again, and cell viability was assessed using a CCK-8 assay kit.

#### 2.2.3. Immunofluorescence

The cells were seeded into a 12-well plate at a density of 1 × 10^5^ cells/mL, with triplicate samples for each group, and incubated overnight. On the following day, the cells were treated with BAP and DIQ either individually or in combination for 8 h. Subsequently, the cells underwent fixation using 4% paraformaldehyde, permeabilization with Triton X-100, and blocking with 5% bovine serum (Solarbio Life Sciences, Beijing, China). Afterward, the cells were then incubated with an Nrf2 antibody and secondary antibody. Ultimately, the cells were visualized under a confocal laser scanning microscope to obtain fluorescent images.

### 2.3. Statistical Analysis

All data were displayed as means ± standard error of the mean (SEM). Statistical analysis was performed using SPSS version 20.0 (Statistical Product and Service Solutions, Inc., Armonk, NY, USA). In this study, Levene’s Test was conducted to assess the homogeneity of variance and the Shapiro–Wilk Test was also employed to evaluate the normal distribution of the data. After verifying that the data satisfied the conditions of homogeneity of variance and normal distribution, differences between groups were conducted using one-way analysis of variance (ANOVA) followed by Duncan’s multiple comparison test. *p* < 0.05 was statistically significant.

## 3. Results

### 3.1. BAPs Influenced Growth Performance in Piglets

The DIQ group showed lower final body weight, average daily weight gain, and daily feed intake from days 21 to 28, along with reduced average daily weight gain and feed intake over the entire period from days 0 to 28 than the control group (*p* < 0.05) (Table 3). Conversely, the BAP group had increased final body weight, daily weight gain, and daily feed intake from days 21 to 28, as well as daily weight gain and feed intake from days 0 to 28, compared to the DIQ group (*p* < 0.05).

### 3.2. BAPs Influenced Intestinal Barrier in Piglets

**Figure 1:**
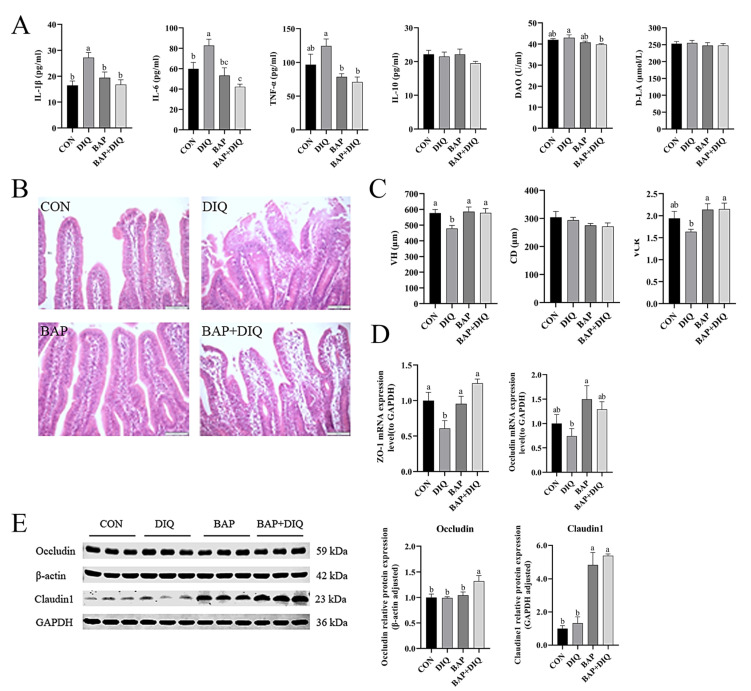
Effects of dietary supplementation with Brown algae polysaccharides on intestinal barrier of piglets. (**A**) Plasma parameters level (n = 6); (**B**) jejunum intestinal morphology (n = 6); (**C**) jejunum villi of crypt height, depth, and villus-to-crypt ratio (n = 6); (**D**) relative mRNA levels of *ZO-1* and *Occludin* (n = 6); (**E**) Occludin and Claudin1 protein abundance (n = 3). Data are presented as means, with error bars representing standard errors. Different letters (a, b, c) in figure indicate significant differences between groups (*p* < 0.05).

There were no significant differences in plasma levels of inflammatory factors, D-LA, and DAO between the BAP group and the control group (*p* > 0.05). However, piglets in the DIQ group showed higher levels of IL-1β and IL-6 than the CON group (*p* < 0.05) (Figure 1A). Piglets in the BAP+DIQ group had lower plasma concentrations of IL-1β, IL-6, TNF-α, and DAO than the DIQ group (*p* < 0.05).

In the CON and BAP groups, piglets’ jejunal villi structures were intact with clear boundaries of the central lacteal. Piglets fed the basal diet and injected with diquat exhibited unclear jejunal villi structures characterized by irregular edges, blurred boundaries of the central lacteal, presence of abundant dispersed material in the intestinal lumen, significant infiltration of inflammatory cells, and proliferation of lymphocytes (Figure 1B). Conversely, in the BAP+DIQ group, jejunal tissue showed irregularly shaped villi with serrated edges, inflammatory cell infiltration, and lymphocyte proliferation, yet maintained intact intestinal morphology with a clear central lacteal. Comparison with the control group revealed no significant differences in jejunal VH, CD, and VCR in piglets of the BAP and BAP+DIQ groups. However, the DIQ group showed a significant reduction in VH compared to the control group (*p* < 0.05) (Figure 1C). The BAP+DIQ group had significantly increased VH and VCR compared to the DIQ group (*p* < 0.05).

Compared with the CON group, the DIQ group displayed a significant reduction in *ZO-1* mRNA expression in the jejunum (*p* < 0.05) (Figure 1D). In contrast, the BAP group exhibited an increase in mRNA expression of both *ZO-1* and *Occludin* compared with the DIQ group (*p* < 0.05). Additionally, the BAP+DIQ group demonstrated an upregulation in *ZO-1* mRNA expression compared to the DIQ group (*p* < 0.05). In terms of protein expression, the BAP group showed an elevation in Claudin1 levels, while the BAP+DIQ group had enhanced protein expression of Occludin and Claudin1 compared to the DIQ group (*p* < 0.05) (Figure 1E).

### 3.3. BAPs Influenced Antioxidant Capacity in Piglets

**Figure 2:**
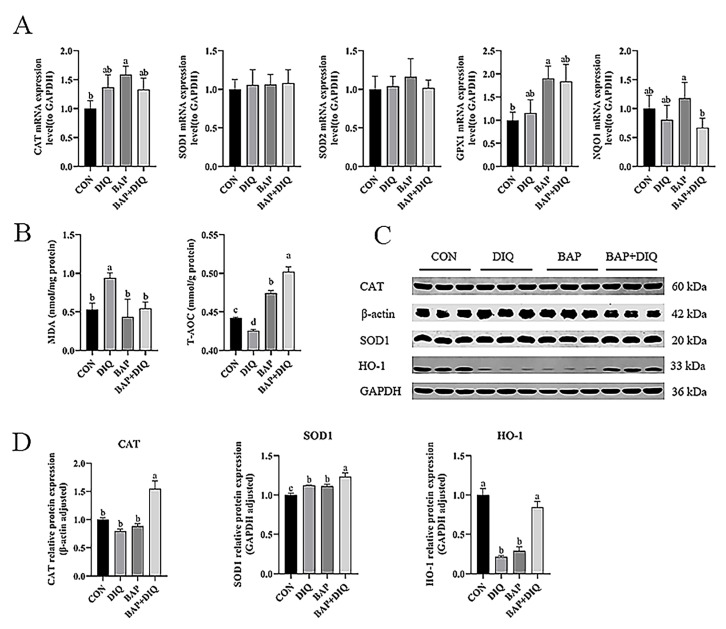
Effects of dietary supplementation with Brown algae polysaccharides on jejunal antioxidant capacity of weaned piglets. (**A**) Relative mRNA levels of *CAT*, *SOD1*, *SOD2*, *GPX1*, and *NQO1* (n = 6); (**B**) jejunal concentrations of MDA, total antioxidation capacity (n = 6); (**C**,**D**) CAT, SOD1, and HO-1 protein abundance (n = 3). Data are presented as means, with error bars representing standard errors. Different letters (a, b, c, d) in figure indicate significant differences between groups (*p* < 0.05).

Feeding BAPs increased mRNA expression of *CAT* and *GPX1* in the jejunum compared with control-fed piglets (*p* < 0.05) (Figure 2A). In contrast, compared to the BAP group, mRNA expression of *NOQ1* in the BAP+DIQ group decreased (*p* < 0.05). Piglets in the DIQ group had higher MDA concentrations and lower T-AOC levels than those in the CON group (*p* < 0.05) (Figure 2B). Conversely, compared with the DIQ group, piglets in the BAP and BAP+DIQ groups exhibited decreased MDA concentrations and elevated T-AOC levels (*p* < 0.05). Compared to the CON group, protein expression of HO-1 in the jejunum of the piglets treated with DIQ decreased (*p* < 0.05) (Figure 2C,D). Compared to the DIQ group, protein expression of CAT, SOD1, and HO-1 in the BAP+DIQ group increased (*p* < 0.05).

### 3.4. BAPs Influenced Barrier Function in IPEC-J2 Cells

**Figure 3:**
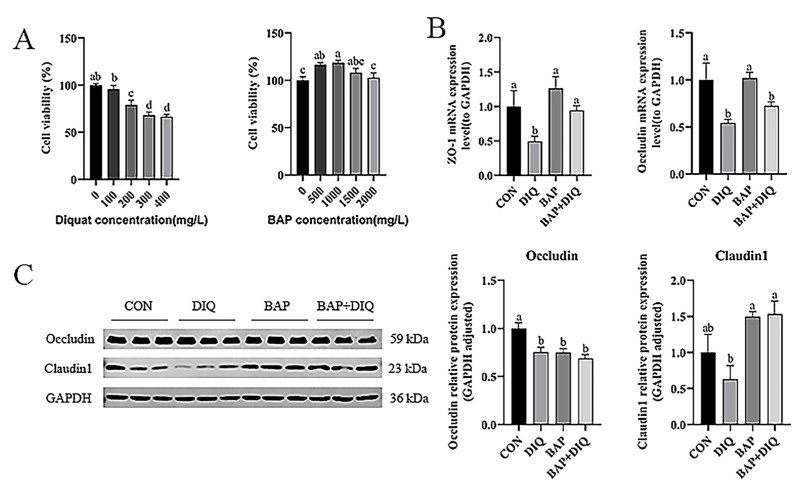
Effects of media supplementation with Brown algae polysaccharides on intestinal barrier of IPEC-J2. (**A**) Cell viability (n = 6); (**B**) relative mRNA levels of *ZO-1* and *Occludin* (n = 6); (**C**) Occludin and Claudin1 protein abundance (n = 3). Data are presented as means, with error bars representing standard errors. Different letters (a, b, c, d) in figure indicate significant differences between groups (*p* < 0.05).

Cells were exposed to different concentrations of DIQ and BAP. As shown in Figure 3A, at a concentration of 100 mg/L, DIQ did not significantly affect the viability of IPEC-J2 cells. However, at concentrations of 200 to 400 mg/L, DIQ reduced the viability of IPEC-J2 cells (*p* < 0.01). Consequently, 200 mg/L of DIQ was selected as the optimal treatment concentration. As depicted in Figure 3A, the cells that were pre-treated with 1000 mg/L BAP had the greatest cell viability. Consequently, 1000 mg/L BAP was selected as the optimal treatment concentration.

In comparison to the CON group, the DIQ group demonstrated a reduction in *ZO-1* and *Occludin* mRNA expression in cells (*p* < 0.05) (Figure 3B). The BAP group had an increase in mRNA expression of *ZO-1* and *Occludin* compared with the DIQ group (*p* < 0.05). The expression of mRNA for *ZO-1* in the BAP+DIQ group was also upregulated compared to the DIQ group (*p* < 0.05). Compared with the CON group, the protein expression of Occludin of the DIQ group was decreased (*p* < 0.05) (Figure 3C). The BAP and BAP+DIQ groups had increased protein expression of Claudin1 compared to the DIQ group (*p* < 0.05).

### 3.5. BAPs Influenced Antioxidant Capacity in IPEC-J2 Cells

**Figure 4:**
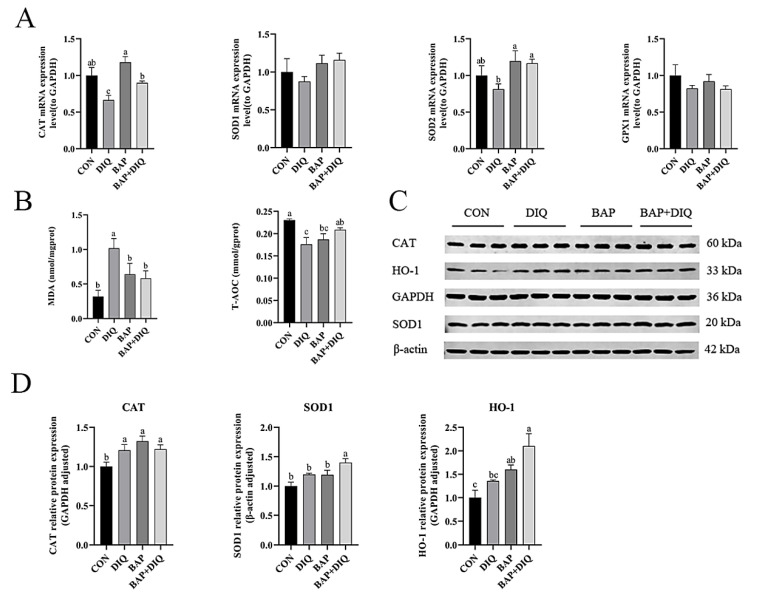
Effects of media supplementation with Brown algae polysaccharides on antioxidant capacity of IPEC-J2. (**A**) Relative mRNA levels of *CAT*, *SOD1*, *SOD2*, *GPX*1 (n = 6); (**B**) Levels of MDA and total antioxidation capacity (n = 6); (**C**,**D**) CAT, SOD1, and HO-1 protein abundance (n = 3). Data are presented as means, with error bars representing standard errors. Different letters (a, b, c) in figure indicate significant differences between groups (*p* < 0.05).

Compared with the CON group, the mRNA expression *CAT* of the *DIQ* group decreased (*p* < 0.05) (Figure 4A). In contrast, compared with the DIQ group, the mRNA expression of *CAT* and *SOD2* increased in the BAP and BAP+DIQ groups (*p* < 0.05). Compared with the CON group, the DIQ group exhibited increased MDA concentration and decreased T-AOC levels (*p* < 0.05) (Figure 4B). Furthermore, the BAP+DIQ group had reduced MDA concentration and increased levels of T-AOC compared to the DIQ group (*p* < 0.05). The protein expression of CAT in cells of the DIQ group increased compared to CON cells (*p* < 0.05) (Figure 4C,D). Compared with the DIQ group, the protein expression of SOD1, and HO-1 in the BAP+DIQ group increased (*p* < 0.05).

### 3.6. BAPs Influenced Nrf2 Signaling Pathway

**Figure 5:**
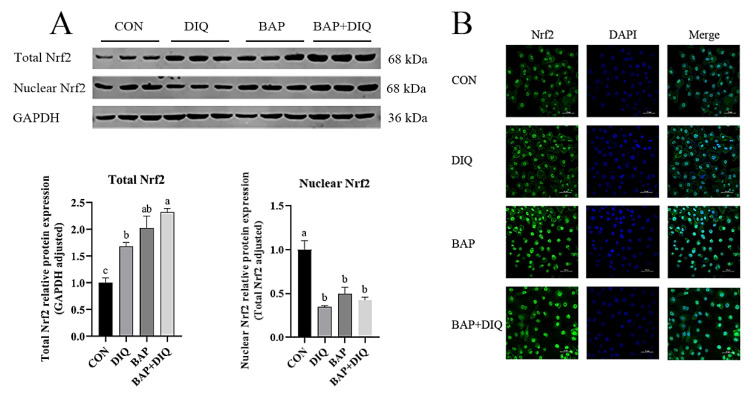
Effects of media supplementation with Brown algae polysaccharides on intestinal Nrf2 signaling pathway. (**A**) Nrf2 protein abundance (n = 3); (**B**) immunofluorescence of Nrf2. Data are presented as means, with error bars representing standard errors. Different letters (a, b, c) in figure indicate significant differences between groups (*p* < 0.05).

Compared with the CON group, the total and nuclear protein expressions of Nrf2 were increased in the BAP and BAP+DIQ treatments (*p* < 0.05) (Figure 5A). Compared with the DIQ group, the total and nuclear protein expressions of Nrf2 were increased in the BAP and BAP+DIQ treatments. Immunofluorescence showed that both DIQ stimulation and BAPs could significantly increase the expression of Nrf2 protein in cells (Figure 5B). Compared to the CON and DIQ groups, the expression of nuclear Nrf2 protein in IPEC-J2 cells was elevated in the BAP and BAP+DIQ groups.

## 4. Discussion

In recent years, seaweeds have attracted attention due to their wide range of biological and physiological activities. Natural antioxidants present in seaweeds are recognized for their high nutritional value, and seaweeds are potentially valuable sources of useful metabolites and bioactive compounds [20]. To explore the mechanism of the antioxidant effect of BAPs, oxidative stress damage following diquat administration was used in in vivo and in vitro models [21]. This study showed that supplementing 0.1% BAPs in piglet fed significantly improved the growth rate from day 0 to day 28. It also increased the total antioxidant capacity levels of antioxidant enzymes, and protein expression for antioxidants in the jejunum. At the same time, BAPs had the same effect on cells in culture, by enhancing cell vitality, antioxidant capacity, and the expression of antioxidant enzymes’ mRNA and proteins. As far as we know, current results are the first to indicate that supplementing BAPs in piglets’ diets can improve growth performance and antioxidant capacity.

Intestinal morphology is considered one of the important parameters to reflect intestinal health and recovery [22]. Oxidative stress can cause damage to the intestinal barrier in piglets due to an imbalance between reactive oxygen species (ROS) and antioxidant systems [23]. The intestinal barrier is a crucial defense line to prevent harmful substances from entering the body. Maintaining integrity of the intestines is vital for the good health of pigs [24]. Levels of DAO and D-lactic acid in the blood are commonly used as useful biomarkers to monitor the integrity of the intestinal barrier [25]. In the present study, BAPs can reduce intestinal permeability by measuring DAO in plasma. Furthermore, adding BAPs to the diet alleviated morphological damage to piglets’ intestines caused by oxidative stress, and increased VH and VCR. Selective permeability of the barrier is achieved through typical structural proteins including ZO-1, Claudin1, and Occludin of epithelial tight junctions [26]. Supplementation with BAPs enhanced the integrity of the jejunal structure, as indicated by increased mRNA and protein expression of ZO-1, Claudin1, and Occludin in jejunal tissue. Cell culture experiments supported these results. These results suggest that supplementing BAPs can enhance intestinal barrier function in piglets.

The production of reactive oxygen species (ROS) leads to oxidative stress, which plays a major role in the inflammatory process [27]. Inflammation is a necessary component of the physiological defense process and is a response to damage caused by oxidative stress [28]. Because oxidative stress initiates inflammation, which in turn contributes to the development of numerous diseases [29], BAPs could alleviate oxidative stress and inhibit DIQ-induced inflammation. Brown algae polysaccharides reduced the levels of the inflammatory factors, IL-1β, IL-6, and TNF-α, in the plasma of piglets, which significantly increased after DIQ exposure. Similarly, fucoidan (a complex sulfated polysaccharide isolated from Brown algae) exhibited effective anti-inflammatory activity [30]. Furthermore, the anti-inflammatory potential of fucoidan extracted from the Brown alga, Turbinaria ornata, was enhanced by enzymatic hydrolysis in in vivo and in vitro models [31]. Therefore, present results suggest that BAPs can protect against DIQ-induced inflammation.

Nrf2 is a key sensor for cellular oxidative stress, functioning as a transcription factor and playing a central role in the regulation of antioxidant enzymes, detoxifying enzymes, and related proteins [32]. Keap1 binds to Nrf2 in the cytoplasm, which promotes Nrf2 ubiquitination, thereby preventing Nrf2 translocation to the nucleus under physiological conditions. When the level of ROS in the cell sharply increases, excess ROS will modify reactive cysteine residues on Keap1, which leads to the dissociation of Keap1 from Nrf2. This dissociation translocates Nrf2 to the nucleus to express its target genes [33]. Different research groups demonstrated that activating the Nrf2 signaling cascade with various pharmacological agents can effectively alleviate oxidative damage in animals. Apple Polyphenols promoted intestinal antioxidant capacity and barrier function via the Nrf2/Keap1 signaling pathway [34]. Disrupting the Keap1-Nrf2 interaction with an inhibitor can activate Nrf2 signaling, thereby alleviating oxidative damage in osteoblasts [35]. However, regulatory effects of BAPs on the Nrf2-ARE pathway have not been reported. In the current experiment, stimulation of IPEC-J2 cells with DIQ resulted in an increase in the expression of total Nrf2 protein. This indicates that, in response to oxidative stress, the cells facilitate the nuclear translocation of Nrf2, thereby mitigating the oxidative damage caused by DIQ. Notably, the levels of both total and nuclear Nrf2 protein expression in the BAP+DIQ group were significantly elevated in comparison to the DIQ group, indicating that BAPs enhanced the expression and nuclear translocation of Nrf2. Nevertheless, the precise mechanisms underlying Nrf2 activation remain to be elucidated. Further research is needed to explore the molecular regulatory mechanisms underlying BAP-induced changes in Nrf2.

## 5. Conclusions

In conclusion, the present research aimed to utilize diquat-induced oxidative stress in piglets and IPEC-J2 cells to investigate the possible protective effect of BAPs on intestinal barrier function and reactive oxygen species levels. The results of this study suggested that adding Brown algae polysaccharides (BAPs) to a corn–soybean-based diet can improve the growth performance and intestinal barrier function of piglets, alleviate oxidative stress, and reduce inflammatory responses induced by DIQ. Brown algae polysaccharides also provide protective effects against diquat-induced oxidative stress in IPEC-J2 cells. Overall, Brown algae polysaccharides play an indispensable role in improving the antioxidant capacity of piglets by activating the Nrf2 signaling pathway. These findings highlight BAP’s potential as a natural feed additive to mitigate oxidative stress and improve overall health in piglets. Further research is warranted to explore BAP as a dietary supplement to support gut health and reduce oxidative stress.

## Figures and Tables

**Table 1:** Composition and nutrient concentration of diets (%, as-fed basis).

Item	Basal Diet	BAP Diet ^a^
Ingredients (%)		
Corn	65.30	65.17
Soybean meal	16.97	17.00
Brown algal polysaccharides	—	0.10
Extruded full-fat soybean	2.00	2.00
Soy protein concentrate	3.00	3.00
Fish meal	3.00	3.00
Whey powder	6.00	6.00
Soybean oil	0.40	0.40
Limestone	0.75	0.75
Dicalcium phosphate	0.80	0.80
NaCl	0.50	0.50
L-lysine HCl	0.45	0.45
L-Threonine	0.16	0.16
L-Tryptophan	0.07	0.07
L-Methionine	0.10	0.10
Premix ^b^	0.50	0.50
Total	100.00	100.00
Nutrient levels ^c^		
DM	88.33	88.34
Ash	4.94	4.61
CP	17.30	17.16
EE	2.75	3.00
NDF	10.62	10.41
ADF	4.82	4.11
GE (MJ/kg)	15.99	16.03

Note: ^a^ Brown algal polysaccharide diet. ^b^ Premix provided per kilogram of feed: vitamin A, 12,000 IU; vitamin B1, 2.5 mg; vitamin B2, 4.0 mg; vitamin B6, 3.0 mg; vitamin B12, 12 μg; vitamin D3, 2500 IU; vitamin E, 30 IU; vitamin K3, 3.0 mg; Nicotinic acid, 40.0 mg; Thiamine, 3.0 mg; Riboflavin, 6.0 mg; D-pantothenic acid, 15.0 mg; Folic acid, 1.2 mg; Biotin, 50.0 μg; Fe, 90.0 mg; Cu, 75.0 mg; Zn, 75.0 mg; Mn, 40.0 mg; I, 0.4 mg; Se, 0.3 mg. ^c^ All nutrient level data are measured values.

**Table 2:** Primers utilized in present study.

Gene	Forward Primer (5′-3′)	Reverse Primer (5′-3′)
*GAPDH*	CGGAGTGAACGGATTTGGC	CACCCCATTTGATGTTGGCG
*ZO-1*	GAAATACCTGACGGTGCTGC	GAGGATGGCGTTACCCACAG
*Occludin*	CAGGTGCACCCTCCAGATTG	TATGTCGTTGCTGGGTGCAT
*CAT*	TGGCTGAGTCCGAAGTCGTC	AAGTAGCCAAAAGCCCCTGCT
*SOD1*	AGGGCACCATCTACTTCGAG	GCACTGGTACAGCCTTGTGT
*SOD2*	AGGCGCTGAAAAAGGGTGAT	GACGGATACAGCGGTCAACT
*GPX1*	ACGCTCGGTGTATGCCTTC	CCCATTCTTGGCATTTTCCTGAT
*NQO1*	GTATAAAGTAGCCGGGCGCT	AGTGCTTTTCTGACCGCCAT

**Table 3:** Effects of dietary supplementation with Brown algae polysaccharides on growth performance of piglets ^1^.

Item	CON	DIQ	BAP	BAP+DIQ	*p*-Value
Initial weight (kg)	8.03 ± 0.38	8.06 ± 0.44	8.24 ± 0.45	8.61 ± 0.43	0.753
Final weight (kg)	22.08 ± 0.125 ^a^	18.45 ± 0.98 ^b^	22.87 ± 0.83 ^a^	20.38 ± 1.13 ^ab^	0.036
21~28 d					
Average daily gain (kg)	0.76 ± 0.07 ^a^	0.36 ± 0.07 ^b^	0.77 ± 0.04 ^a^	0.43 ± 0.07 ^b^	<0.001
Average daily intake (kg)	1.27 ± 0.06 ^a^	0.81 ± 0.05 ^b^	1.27 ± 0.09 ^a^	0.76 ± 0.07 ^b^	<0.001
Feed: Gain	1.71 ± 0.09	2.04 ± 0.16	1.64 ± 0.05	1.93 ± 0.23	0.228
0~28 d					
Average daily gain (kg)	0.50 ± 0.04 ^ac^	0.39 ± 0.02 ^b^	0.52 ± 0.02 ^a^	0.42 ± 0.04 ^bc^	0.016
Average daily intake (kg)	0.87 ± 0.04 ^a^	0.70 ± 0.05 ^b^	0.89 ± 0.05 ^a^	0.72 ± 0.05 ^b^	0.021
Feed: Gain	1.75 ± 0.07	1.75 ± 0.07	1.69 ± 0.03	1.74 ± 0.04	0.855

Note: ^1^ Average daily gain (ADG) = daily gain/day; average daily feed intake (ADFI) = daily feed intake/day; feed conversion ratio (F, G) = piglet feed intake/piglet weight gain (n = 6). CON group, piglets without BAP or DIQ treatment; DIQ group, piglets treated with DIQ; BAP group, piglets treated with BAP; BAP+DIQ group, piglets pre-treated with BAP and subsequently treated with DIQ. ^a,b,c^ Mean values within row with unlike superscript letters are different (*p* < 0.05).

## Data Availability

The data used to support the findings of this study are available from the corresponding author on reasonable request.

## Supplementary Materials

The following supporting information can be downloaded at: https://www.mdpi.com/article/10.3390/ani15040559/s1, Antibodies information; Western blotting.
