# Supplementary material for: Brown Algae Polysaccharides Alleviate Diquat-Induced Oxidative Stress in Piglets and IPEC-J2 Cells via Nrf2/ARE Signaling Pathway
Source: Animals (Basel). 2025 Feb 14;15(4):559. doi: 10.3390/ani15040559 (PMC11852254; doi:10.3390/ani15040559)
Supplement: Supplementary file 1 [file animals-15-00559-s001.zip › animals-3440060-supplementary.pdf]

Antibodies information

Primary antibodies include Occludin (Cat: 66378-1-IG, Proteintech, Rosemont, IL, USA), Claudin (Cat: 28674-1-AP, Proteintech, Rosemont, IL, USA), CAT (Cat: 21260-1-AP, Proteintech, Rosemont, IL, USA), SOD1 (Cat: 10269-1-AP, Proteintech, Rosemont, IL, USA), HO-1 (Cat: 10701-1-AP, Proteintech, Rosemont, IL, USA), Nrf2 (Cat: 16396-1-AP, Proteintech, Rosemont, IL, USA), β-actin (Cat: 66009-1-IG, Proteintech, Rosemont, IL, USA), GAPDH (Cat: 60004-1-IG, Proteintech, Rosemont, IL, USA). Secondary antibodies include anti-rabbit immunoglobulin G (IgG) (Cat:5151S, Cell Signaling Technology, Danvers, MA, USA), and Anti-mouse immunoglobulin G (IgG) (Cat:5257P, Cell Signaling Technology, Danvers, MA, USA).

Western blotting

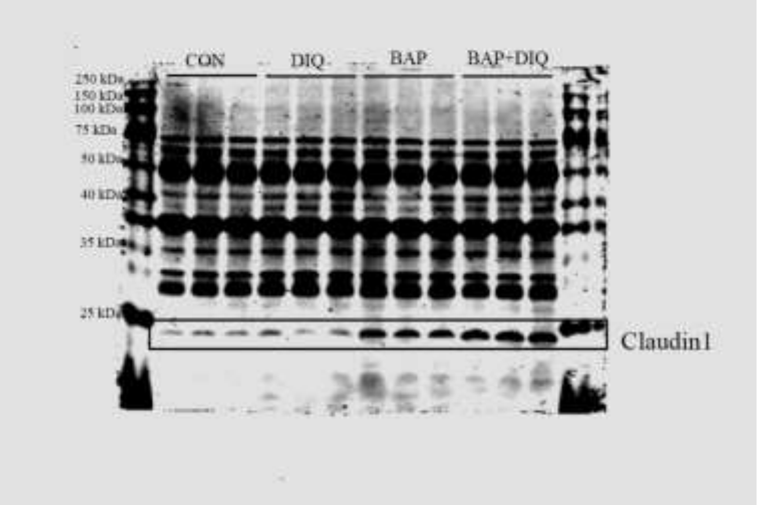

The figure above is used for Figure 1E in the article. Gel membrane and protein band of Claudin1 (23 kDa) in jejunum tissue of piglets. CON means CON group; DIQ means DIQ group; BAP means BAP group, BAP+DIQ means BAP+DIQ group.

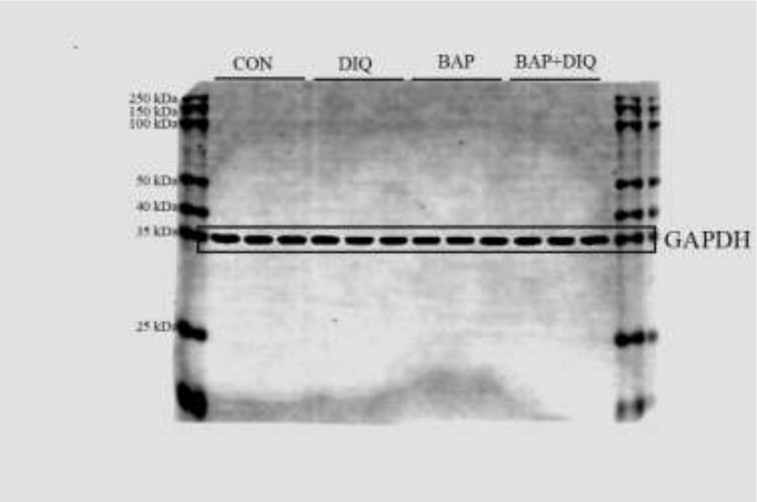

The figure above is used for Figure 1E in the article. Gel membrane and protein band of GAPDH (36 kDa) as the loading control for Claudin1 in jejunum tissue of piglets. CON means CON group; DIQ means DIQ group; BAP means BAP group, BAP+DIQ means BAP+DIQ group.

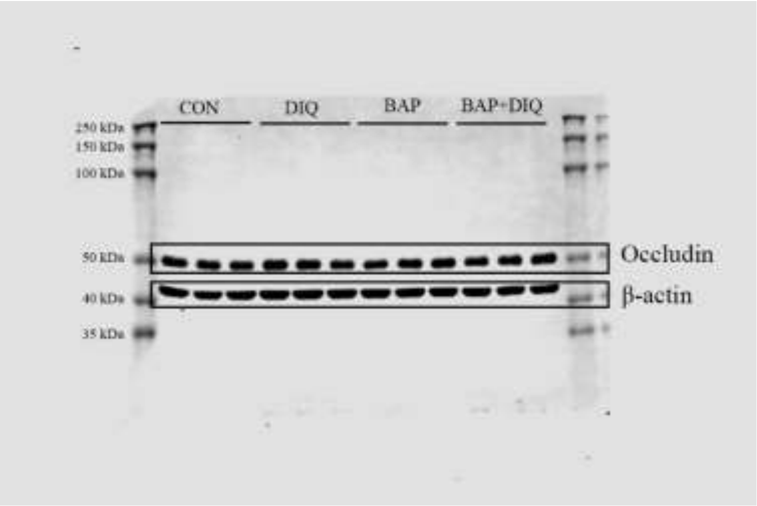

The figure above is used for Figure 1E in the article. Gel membranes and protein bands of Occludin (59 kDa) and β-actin (42 kDa) as the loading control for Occludin in jejunum tissue of piglets. CON means CON group; DIQ means DIQ group; BAP means BAP group, BAP+DIQ means BAP+DIQ group.

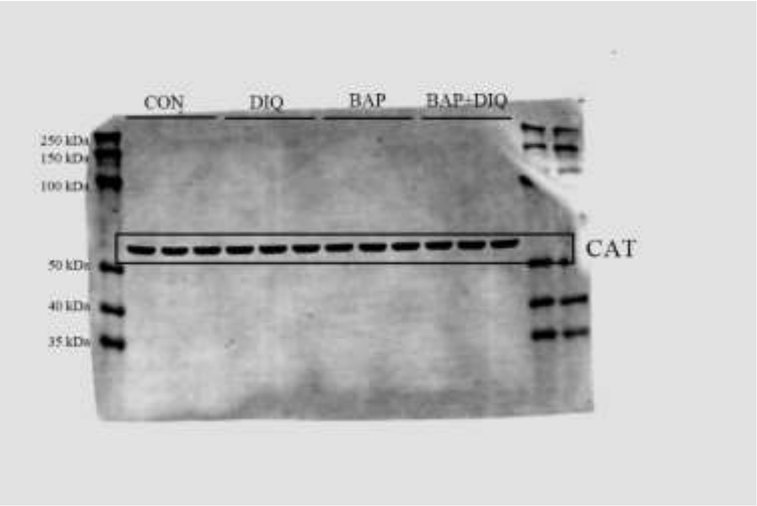

The figure above is used for Figure 2C in the article. Gel membrane and protein band of CAT (60 kDa) in jejunum tissue of piglets. CON means CON group; DIQ means DIQ group; BAP means BAP group, BAP+DIQ means BAP+DIQ group.

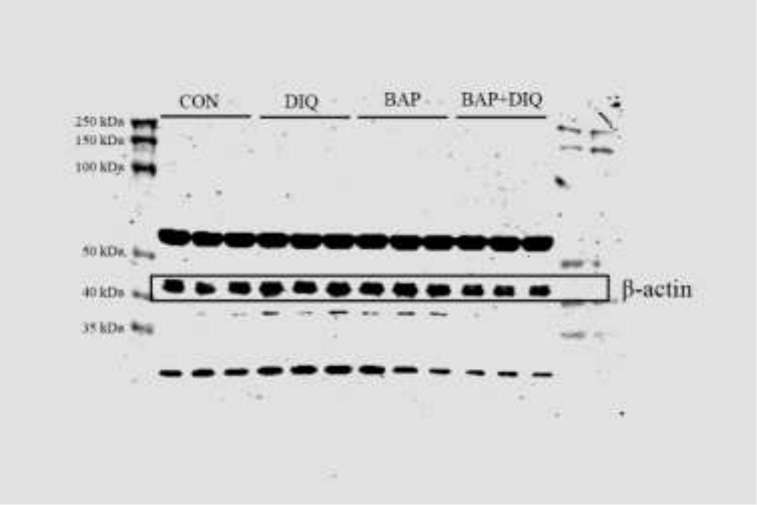

The figure above is used for Figure 2C in the article. Gel membrane and protein band of β-actin (42 kDa) as the loading control for CAT in jejunum tissue of piglets. CON means CON group; DIQ means DIQ group; BAP means BAP group, BAP+DIQ means BAP+DIQ group.

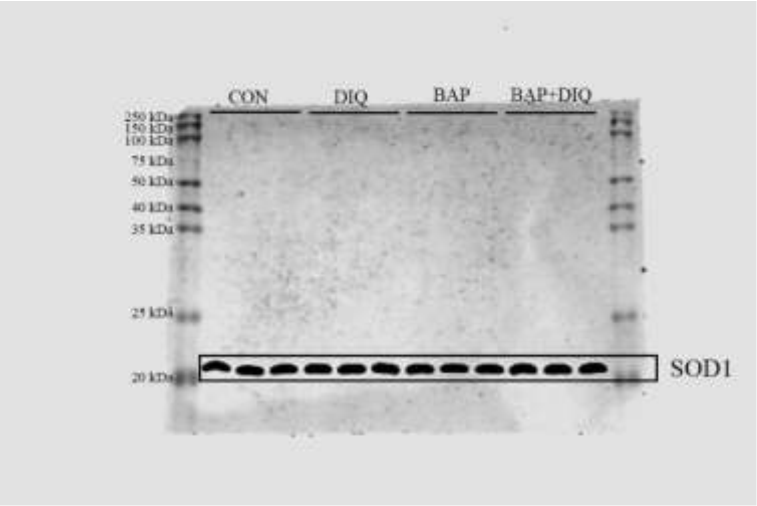

The figure above is used for Figure 2C in the article. Gel membrane and protein band of SOD1 (20 kDa) in jejunum tissue of piglets. CON means CON group; DIQ means DIQ group; BAP means BAP group, BAP+DIQ means BAP+DIQ group.

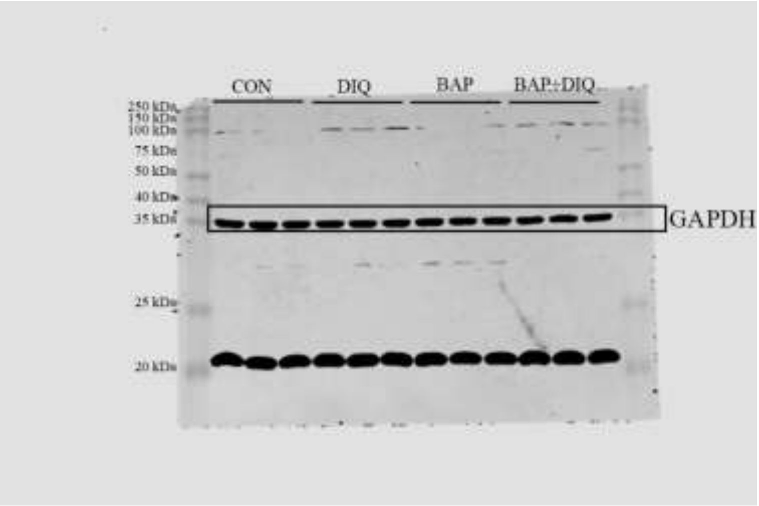

The figure above is used for Figure 2C in the article. Gel membrane and protein band of GAPDH (36 kDa) as the loading control for SOD1 in jejunum tissue of piglets. CON means CON group; DIQ means DIQ group; BAP means BAP group, BAP+DIQ means BAP+DIQ group.

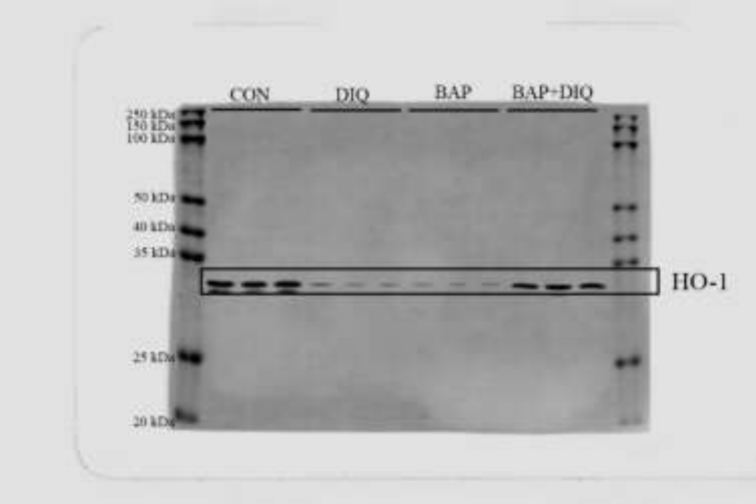

The figure above is used for Figure 2C in the article. Gel membrane and protein band of HO-1 (33 kDa) in jejunum tissue of piglets. CON means CON group; DIQ means DIQ group; BAP means BAP group, BAP+DIQ means BAP+DIQ group.

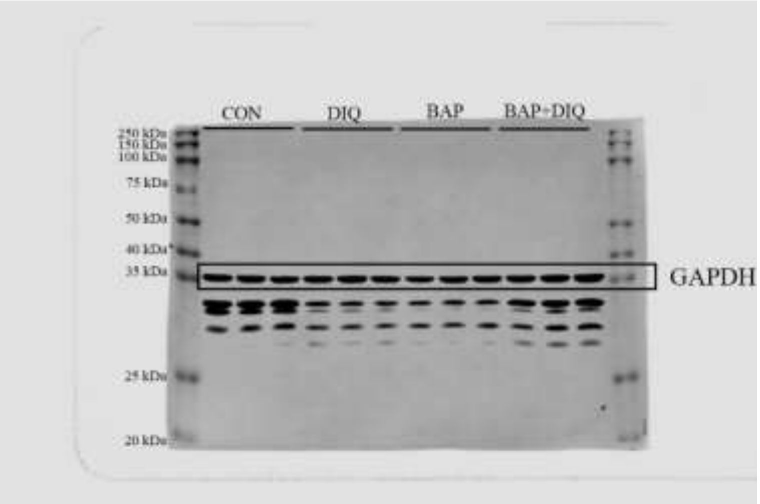

The figure above is used for Figure 2C in the article. Gel membrane and protein band of GAPDH (36 kDa) as the loading control for HO-1 in jejunum tissue of piglets. CON means CON group; DIQ means DIQ group; BAP means BAP group, BAP+DIQ means BAP+DIQ group.

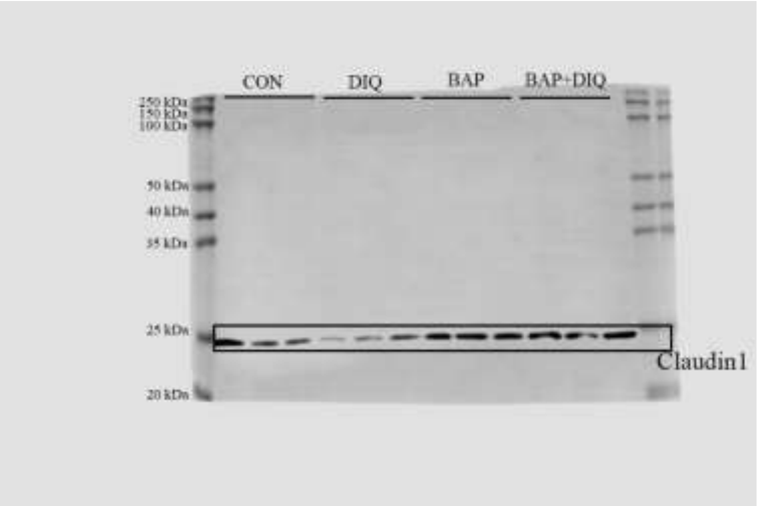

The figure above is used for Figure 3C in the article. Gel membrane and protein band of Occludin (23 kDa) in IPEC-J2 cells. CON means CON group; DIQ means DIQ group; BAP means BAP group, BAP+DIQ means BAP+DIQ group.

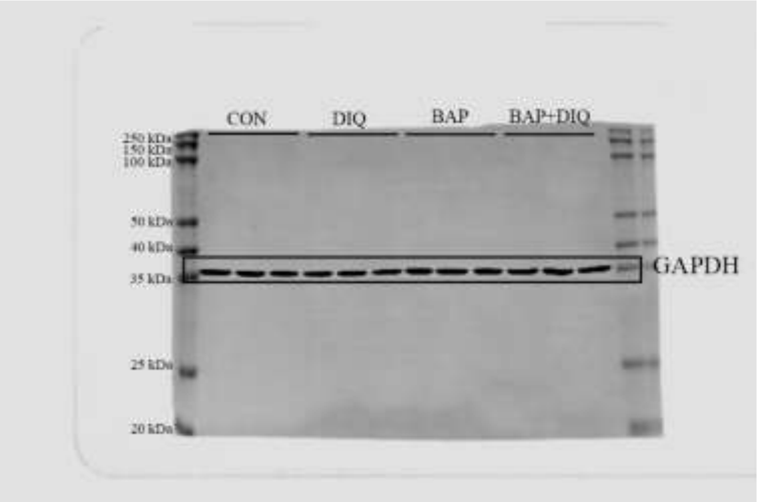

The figure above is used for Figure 3C in the article. Gel membrane and protein band of GAPDH (36 kDa) as the loading control for Claudin1 in IPEC-J2 cells. CON means CON group; DIQ means DIQ group; BAP means BAP group, BAP+DIQ means BAP+DIQ group.

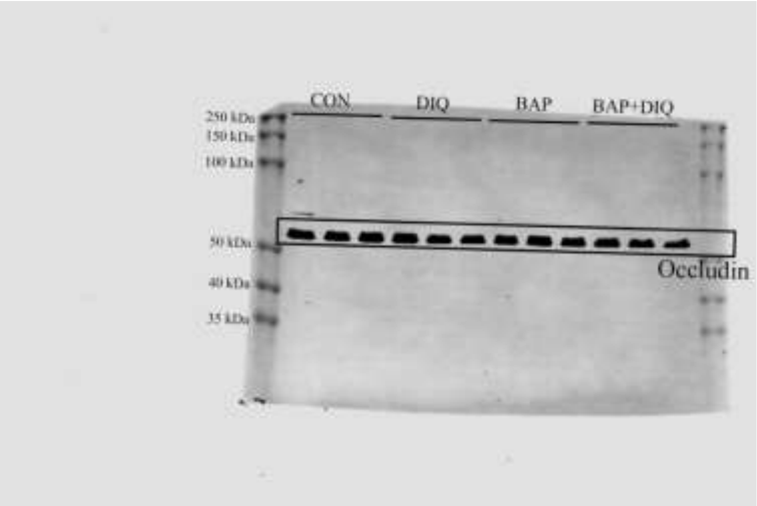

The figure above is used for Figure 3C in the article. Gel membrane and protein band of Occludin (59 kDa) in IPEC-J2 cells. CON means CON group; DIQ means DIQ group; BAP means BAP group, BAP+DIQ means BAP+DIQ group.

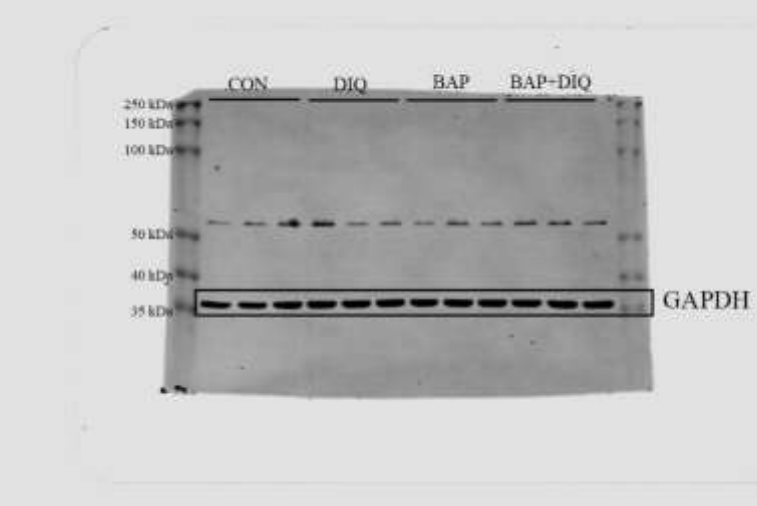

The figure above is used for Figure 3C in the article. Gel membrane and protein band of GAPDH (36 kDa) as the loading control for Occludin in IPEC-J2 cells. CON means CON group; DIQ means DIQ group; BAP means BAP group, BAP+DIQ means BAP+DIQ group.

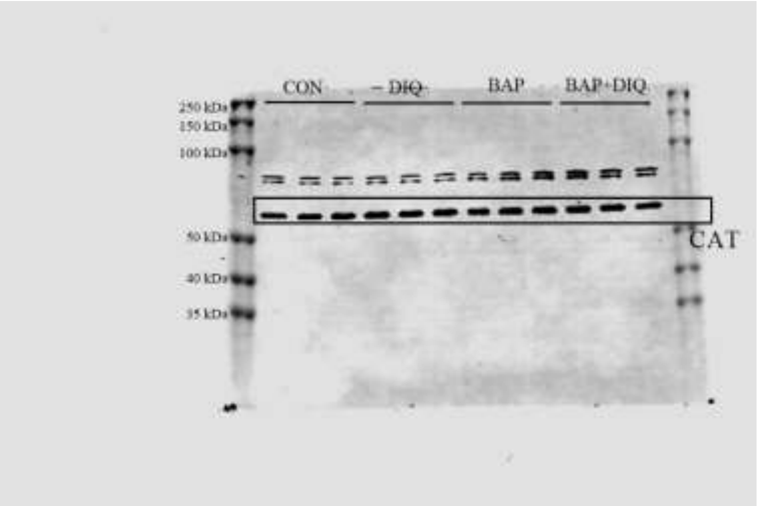

The figure above is used for Figure 4C in the article. Gel membrane and protein band of CAT (60 kDa) in IPEC-J2 cells. CON means CON group; DIQ means DIQ group; BAP means BAP group, BAP+DIQ means BAP+DIQ group.

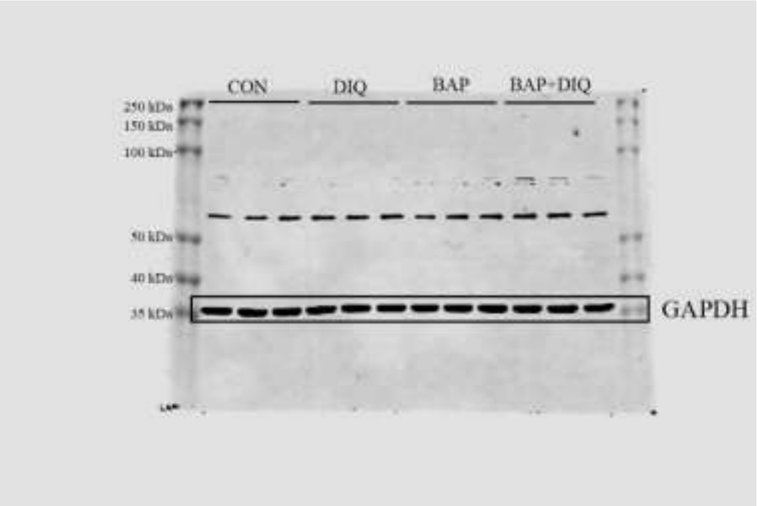

The figure above is used for Figure 4C in the article. Gel membrane and protein band of GAPDH (36 kDa) as the loading control for CAT in IPEC-J2 cells. CON means CON group; DIQ means DIQ group; BAP means BAP group, BAP+DIQ means BAP+DIQ group.

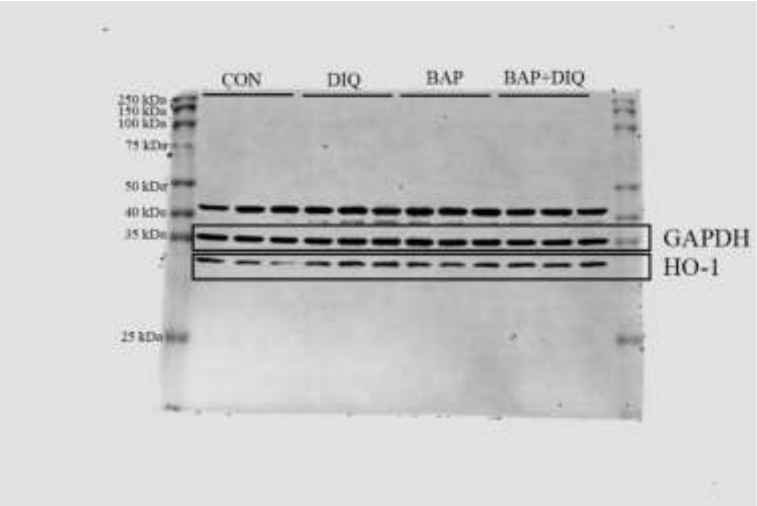

The figure above is used for Figure 4C in the article. Gel membranes and protein bands of HO-1 (33 kDa) and GAPDH (36 kDa) as the loading control for HO-1 in IPEC-J2 cells. CON means CON group; DIQ means DIQ group; BAP means BAP group, BAP+DIQ means BAP+DIQ group.

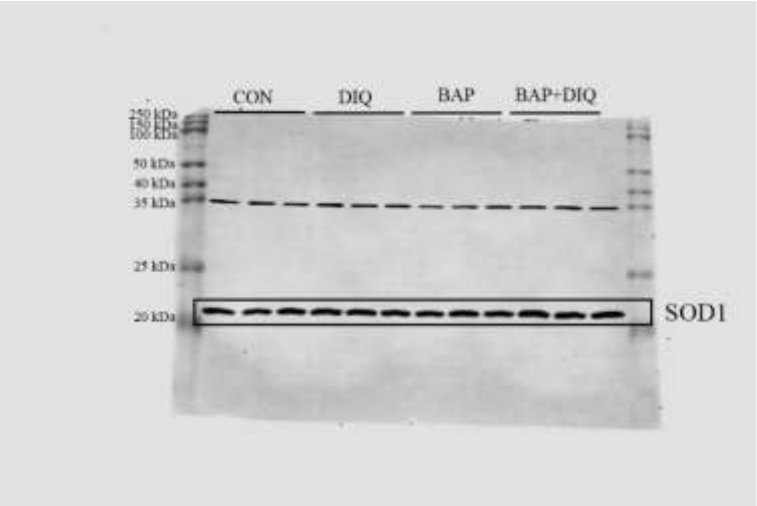

The figure above is used for Figure 4C in the article. Gel membrane and protein band of SOD1 (20 kDa) in IPEC-J2 cells. CON means CON group; DIQ means DIQ group; BAP means BAP group, BAP+DIQ means BAP+DIQ group.

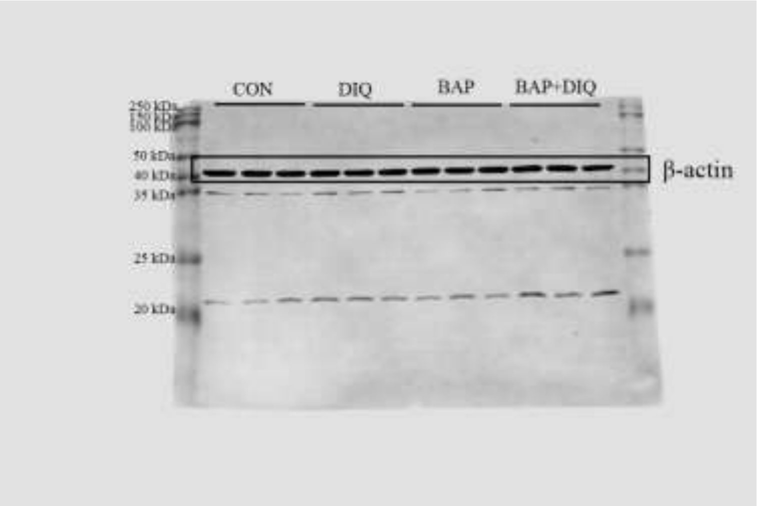

The figure above is used for Figure 4C in the article. Gel membrane and protein band of β-actin (42 kDa) as the loading control for SOD1 in IPEC-J2 cells. CON means CON group; DIQ means DIQ group; BAP means BAP group, BAP+DIQ means BAP+DIQ group.
